# ING Proteins and Neural Development in Newborns from Alcohol- and/or Drug-Abusing Pregnant Women

**DOI:** 10.3390/ijms27104383

**Published:** 2026-05-14

**Authors:** Sergio Terracina, Luigi Tarani, Mauro Ceccanti, Giovanna Blaconà, Marco Fiore, Giampiero Ferraguti

**Affiliations:** 1 UOC Clinical Biochemistry and Molecular Biology, Sant’Andrea University Hospital, 00189 Rome, Italy; sergio.terracina@uniroma1.it; 2 Department of Maternal Infantile and Urological Sciences, Sapienza University of Rome, 00185 Rome, Italy; luigi.tarani@uniroma1.it; 3 Società Italiana per il Trattamento dell’Alcolismo e le sue Complicanze (SITAC), 00185 Rome, Italy; mauro.ceccanti@fondazione.uniroma1.it; 4 Department of Experimental Medicine, Sapienza University of Rome, 00185 Rome, Italy; giovanna.blacona@uniroma1.it; 5 Institute of Biochemistry and Cell Biology (IBBC-CNR), Department of Sensory Organs, Sapienza University of Rome, 00185 Rome, Italy

**Keywords:** alcohol, epigenetic, inhibitor of growth proteins, neurodevelopment, oxidative stress, substance abuse

## Abstract

Maternal consumption of alcohol and drugs during pregnancy can compromise neural development with long-lasting impact on individuals’ health. The inhibitor of growth (ING) family of proteins is an epigenetic regulator that plays a central role in fetal brain development, contributing to neural stem cell maintenance, neuronal differentiation, and the regulation of genes involved in brain morphogenesis. Given the susceptibility of the developing nervous system to epigenetic dysregulation induced by alcohol and drugs, this narrative study aims to summarize literature evidence with the hypothesis that ING proteins may represent a critical but understudied mechanistic link between maternal substance dependence and adverse neurodevelopmental outcomes in newborns. We conducted a comprehensive literature search across three databases (PubMed, Scopus, and Web of Science) up to February 2026 to identify relevant studies. Search terms included combinations of “ING proteins”, “neural development”, “alcohol”, “drugs”, “epigenetic”, “oxidative stress” and “neuroinflammation”. The inclusion criteria were limited to original studies published in English that examined neural development in newborns; the exclusion criteria encompassed non-English publications, letters, editorials, and case reports, and those not directly addressing the specified topics. We identified 55 papers; six were excluded per the exclusion criteria, leaving 49 works discussed in this review. ING proteins are epigenetic regulators essential for embryonic and neural development, including neural stem cell fate and neurogenesis, while substances of abuse are disruptors of the essential pathways necessary for the right fetal brain development. Furthermore, substance abuse creates oxidative stress environments and activates pathways that require ING-mediated chromatin regulation. ING proteins likely act as mediators linking oxidative stress, neuroinflammation, and transcriptional reprogramming in the developing brain. Meanwhile, alcohol and drugs induce epigenetic reprogramming that may disrupt ING-mediated chromatin control. There is little evidence directly linking prenatal exposure (e.g., alcohol and drugs) to ING changes during fetal development. However, we hypothesize that ING proteins function as epigenetic stress response regulators whose disruption by oxidative stress, inflammation, and chromatin alterations induced by prenatal alcohol or drug exposure may contribute to impaired fetal neurodevelopment. Although direct experimental evidence remains limited, this could be a promising and relatively unexplored research area.

## 1. Introduction

Neural development during the perinatal period is a highly coordinated process regulated by genetic, epigenetic, and environmental factors [1,2]. Disruptions occurring in utero can have profound and lasting effects on fetal brain structure and function, often manifesting as cognitive deficits, behavioral abnormalities, or neurodevelopmental disorders later in life [3]. It is estimated that (despite important differences based on age, geolocation, and socioeconomical disadvantage) up to 30% of pregnant women smoke and/or use cannabis, 15% drink alcohol, and up to 21% (highest risk in teenagers) use other illicit drugs worldwide [4,5]. Furthermore, exposure to more than one potentially harmful substance or drug is common [6]. In fact, among the most impactful environmental insults on fetal health are maternal consumption of alcohol and drugs during pregnancy, which can compromise neurogenesis, synaptogenesis, neuronal migration, and circuit maturation.

Prenatal alcohol exposure is a well-established teratogen and the leading preventable cause of neurodevelopmental impairment worldwide, causing a spectrum of outcomes collectively defined as fetal alcohol spectrum disorders (FASDs), encompassing structural multiorgan anomalies, altered neuronal differentiation, and long-term cognitive and behavioral impairments [7,8]. Similarly, prenatal exposure to substances such as opioids, cocaine, methamphetamine, and cannabis has been linked to aberrant neurodevelopmental trajectories, including disturbances in neuronal proliferation, epigenetic reprogramming, and impaired maturation of neural networks. Epigenetic regulators are emerging as key mediators of how environmental stressors influence fetal brain development [9,10,11]. Among these, the inhibitor of growth (ING) family of proteins plays a central role in chromatin remodeling, DNA repair, apoptosis, and transcriptional regulation [12,13]. ING proteins modulate histone acetylation states and interact with key signaling pathways essential for neurodevelopment [14]. Preclinical evidence suggests that ING proteins contribute to neural stem cell maintenance, neuronal differentiation, and the regulation of genes involved in brain morphogenesis [15].

Despite these insights, their involvement in human neonatal brain development, particularly under conditions of prenatal toxic exposure, remains largely unexplored. Given the susceptibility of the developing nervous system to epigenetic dysregulation induced by alcohol and drugs, ING proteins may represent a critical but understudied mechanistic link between maternal substance dependence and adverse neurodevelopmental outcomes in newborns.

This narrative review aims to summarize the literature evidence on the potential association between ING protein expression and neurodevelopmental alterations in babies born to mothers dependent on alcohol and/or drugs, thereby exploring a possible mechanistic link between prenatal toxic exposure and impaired neural development.

## 2. Results of the Reviewed Studies

### 2.1. ING Family: Biological Role and Functions

Although ING proteins have been primarily studied in cancer biology, their roles in chromatin regulation, stress responses, and stem cell biology make them highly relevant to neurodevelopmental processes, particularly in the context of prenatal environmental insults. The ING proteins constitute a family of type II tumor suppressors with highly conserved structural, functional, and evolutionary characteristics, involved in the regulation of cell cycle progression, DNA repair, and chromatin remodeling through their interactions with histone-modifying complexes (see Table 1 for ING family proteins, molecular mechanisms and biological function) [16]. A total of five members have been included in mammals for this family, each producing several isoforms.

Among the shared hallmark features between the ING proteins the most important are (1) the highly conserved C-terminal plant homeodomain (PHD) finger, which binds to the histone mark, trimethylated lysine 4 on histone H3 (H3K4me3), enabling ING proteins to localize chromatin-modifying complexes to the right genomic regions; (2) the capacity to target histone-modifying complexes as non-enzymatic regulatory subunits within either or histone deacetylase-HDAC (ING1 and ING2) and histone acetyltransferase-HAT (ING3, ING4 and ING5) complexes to active chromatin using the PHD domain; (3) their action as tumor suppressors and participation in the DNA damage response (DDR); and their regulation by stress-induced nuclear translocation (e.g., in the case of DNA damage), often via phosphorylation-dependent nuclear localization sequence (NLS) exposure to enable rapid access to chromatin.

Briefly, ING1 was discovered in 1996 and represents the prototypical and most extensively characterized member [17]. Its human gene is located on chromosome 13q34 and encodes multiple splice variants, of which ING1b is the most abundant and functional isoform [18]. ING1b primarily associates with mSin3A–HDAC1/2 complexes to promote histone deacetylation, repressing transcription [19]. It stabilizes and acetylates p53, inducing growth arrest, apoptosis and senescence, and it is a key controller of replication stress via proliferating cell nuclear antigen (PCNA) binding [20]. ING2a represents the ING2 main isoform. Similar to ING1, the second protein of this family is associated with HDAC1/2 and is a strong activator of p53, but it also interacts with p300, allowing dual HAT/HDAC roles and regulation of replicative senescence [14,21,22]. Uniquely among ING proteins, it has roles in metabolism and lipid homeostasis [23]. It plays a major role in regulating the cytoskeleton and cell migration/invasion. It is often downregulated in various carcinomas, making it a potential biomarker or therapeutic target in some cases [24,25]. ING3 has a simpler architecture comprising only one main isoform and is a core subunit of the TIP60/NuA4 HAT complex [26,27]. It is involved in DNA double-strand break repair (ATM/ATR and homology-directed repair (HDR) pathway), chromatin opening, transcriptional activation and inhibition of the Wnt/β-catenin pathway [28]. Knockout studies showed its essentiality for embryonic development (early lethality), highlighting its potential relevance in neurodevelopmental contexts [29]. ING4 is considered the anti-inflammatory and anti-angiogenic member [30,31]. It has one major isoform and some minor variants arising from alternative start sites. It interacts primarily with the HBO1–HAT chromatin complex and with MOZ/MORF HAT complexes (which are critical for H3/H4 acetylation and DNA replication licensing) [32,33]. It is a strong inhibitor of NF-κB signaling (RelA), HIF-1α-mediated angiogenesis and inflammatory cytokine expression. ING4 promotes DNA repair and maintains chromatin stability and is frequently lost in gliomas, melanoma, and breast cancer. Finally, ING5 has only one main isoform, interacting with the HBO1–HAT complex and the MOZ/MORF HAT complex, as ING4 does [34,35]. ING5 differentially regulates protein lysine acetylation and promotes p300 autoacetylation [36]. ING5 knockout may have postpartum effects on stem cell maintenance [29]. It generally controls DNA replication initiation (MCM complex), S-phase progression, and chromatin assembly during replication. Still, strangely, it has been reported to have both tumor suppressive and proliferative roles depending on the context [37,38]. ING5 is essential for stem cell proliferation, particularly in epithelial tissues, and is involved in neural progenitor maintenance, suggesting a role in brain development [39].

Taken together, these features indicate that ING proteins are not only key regulators of genome stability and chromatin dynamics, but also potentially critical modulators of cellular processes that are fundamental for normal neurodevelopment. This provides the biological rationale for exploring their role in the context of prenatal exposure to alcohol and drugs. As we will further explore in more detail below, ING proteins are also most relevant in contexts involving oxidative stress, inflammation, epigenetic regulation and neurodevelopmental outcomes.

### 2.2. Oxidative Stress

Maternal oxidative stress during pregnancy is a major determinant for offspring neurodevelopment and has been associated with several neurodevelopmental disorders [40,41,42]. Cells are continuously exposed to various endogenous or exogenous factors that can dangerously threaten the integrity of genomic asset composition, among them being oxidative damage, which the human organism produces physiological defenses against; when these defenses fail, disorders and disease can take over [43]. Sources of increased oxidative damage are both endogenous (mitochondrial respiration, NADPH oxidases, xanthine oxidase, cytochrome P450 enzymes, peroxisomes, and inflammation) and exogenous (UV and X-ray radiation, pollutants, smoke, alcohol, and pesticides) so that oxidative stress occurs when reactive oxygen species (ROS) and reactive nitrogen species (RNS) exceed the body’s ability to neutralize them [44,45]. Major ROS and RNS include superoxide anion (O_2_•^−^), hydrogen peroxide (H_2_O_2_), which crosses membranes, hydroxyl radical (•OH), which is the most destructive among the ROS via the Fenton reaction, peroxynitrite (ONOO^−^) and nitric oxide (NO•). ROS react with all biomolecules, including DNA (especially with mitochondrial DNA), proteins and lipids, with an increased risk of carcinogenesis, neurodegenerative diseases and atherosclerosis [46].

Mitochondria are particularly vulnerable, as ROS damage sets up a vicious cycle that generates more ROS. Our body uses three lines of defense against ROS damage: enzymatic antioxidants, non-enzymatic antioxidants (chemical scavengers) and cellular and molecular stress responses. Enzymatic antioxidants are considered the primary defense and include superoxide dismutases (SOD1, SOD2, and SOD3), which convert O_2_•^−^ into H_2_O_2_; catalase, which converts H_2_O_2_ into H_2_O + O_2_; glutathione (GSH) peroxidases, which reduce H_2_O_2_ and lipid peroxides using GSH; and peroxiredoxins and the thioredoxin system [47]. Non-enzymatic antioxidants include endogenous (GSH, uric acid, coenzyme Q10, lipoic acid, and melatonin) and dietary (ascorbate, α-tocopherol, carotenoids, flavonoids, polyphenols, selenium, and zinc) products. Oxidative stress induces cellular and molecular responses regulated by various pathways and systems, depending on the location and extent of damage [nuclear factor erythroid 2-related factor 2 (NRF2) pathway, DNA repair systems, proteostasis, mitophagy, and inflammatory modulation].

It has been demonstrated that most addictive substances (ethanol, methamphetamine, cocaine, opioids, etc.) increase ROS and RNS while compromising antioxidant defenses [48]. These substances increase oxidative markers such as malondialdehyde, thiobarbituric acid-reactive substances, and lipid peroxidation, while reducing enzymatic antioxidant levels (superoxide dismutase, glutathione peroxidases, and catalase) and the total antioxidant capacity [49,50,51]. Interestingly, a recent article evaluated the blood concentration of several enzymatic antioxidants during distinct phases of alcohol and opioid dependency (intoxication and withdrawal), finding that the oxidative stress associated with these two forms of dependency may involve different redox mechanisms leading to diverse possible predispositions to neurological diseases [47].

In particular, plasma total superoxide dismutase is significantly increased only in alcohol dependence, suggesting a stronger activation of first-line antioxidant defenses regardless of intoxication or withdrawal. Meanwhile, the opioid transition from intoxication to withdrawal is associated with an increase in catalase activity and reduced activity of the glutathione system, which is a major neuroprotective component. Furthermore, the literature evidence shows that oxidative stress associated with substance abuse and dependence, both during pregnancy and preconception, is a major cause of neurodevelopmental diseases [52].

Methamphetamine elevates ROS and triggers ROS-dependent induction of pro-inflammatory cytokine genes (IL-1β and TNF-α); in individuals with HIV, methamphetamine can synergize with HIV-1 Tat to impair antioxidant defenses, which further enhances NF-κB activation [53]. Because the upregulated gene modules are enriched for targets linked to TBP, ING4, and IRF2, these findings suggest that oxidative stress-driven NF-κB signaling may converge with ING4-associated chromatin control to coordinate the downstream transcriptional response [53].

As ING proteins are epigenetic readers that recruit chromatin modifiers and thereby influence transcriptional programs, they play an important role in processes activated by genotoxic or redox stress. Indirect evidence suggests a direct link where oxidative stress represents the primary upstream insult, while ING proteins act as stress response modulators. The majority of ROS are generated by leakage of electrons in the mitochondrial respiratory chain, contributing to mitochondrial and cellular damage. Meanwhile, ING1 has been shown to translocate to mitochondria in response to apoptotic stimuli and to modulate mitochondrial membrane potential and pro-apoptotic signaling (Bax and cytochrome-c), connecting ING1 function to mitochondrial stress and ROS-related apoptosis [54,55]. Conversely, the loss of ING2, which also localizes to mitochondria in a redox-sensitive manner and is involved in mitochondrial respiration, seems to confer protection against mitochondrial respiratory chain inhibition [56]. Loss of mitochondrial ING2 alters mitochondrial ultrastructure, reduces oxidative phosphorylation activity and lowers mitochondrial ROS production. ING3 is required for proper ataxia–telangiectasia mutated (ATM) signaling and DNA repair responses, pathways that are activated by oxidative DNA damage [57,58]. All ING proteins except ING3 have been observed to coimmunoprecipitate with p53 and modulate p53 transcriptional responses, including those that regulate antioxidant genes and ROS levels [59]. These results suggest that the ING family may participate in redox mechanisms and so play a role in neurodevelopmental oxidative stress-related diseases.

### 2.3. Inflammation

Maternal health and immune activation, whether from infection, systemic inflammation, stress, or other immune-altering conditions, appear to alter fetal brain development significantly [60,61]. In fact, they are associated with a higher risk of neurodevelopmental disorders in human offspring. This association involves complex interactions among immune signaling, placental biology, microglia and gene expression regulation. In fact, inflammatory molecules can cross the placenta and act at the placenta, reducing nutrient exchange and altering the signaling environment. Meanwhile, the function of brain microglia, immune cells, may be affected by inflammatory molecules, disrupting synaptic development. Furthermore, maternal dysregulated immune pathways have been linked to epigenetic changes associated with neurodevelopmental diseases.

Prenatal exposure to alcohol, cocaine and other substances causes abnormalities in fetal brain development, which are linked to later development of neurobehavioral and cognitive dysfunction [7,62,63]. Significant interference with the normal development and maturation of the brain due to the exposure to these substances has been associated with neuroinflammatory signaling load. In fact, chronic alcohol and drug consumption causes both ROS damage and neuroinflammation in the brain [48,64]. A recent article found that the neuroinflammatory environment caused by cocaine exposure has parallels with the neurodevelopmental signaling perturbations associated with opioid and cocaine exposure in utero [62]. In fact, this environment seems to be produced through activation of astrocyte-like cells (AS2 cluster) due to upregulation in cytokine neuroinflammatory signaling and oxidative stress in the developing cerebral organoids following substance exposure, leading to injury/damage responses. Similarly, alcohol abuse during pregnancy increases maternal circulatory cytokines (IL-6 and TNF-α); these molecules (together with increased oxidative stress) lead to placental dysfunction, causing impaired nutrient and oxygen delivery, and, upon crossing this site, transfer to the fetus, creating an abnormal inflammatory milieu in the brain [65,66]. Furthermore, alcohol causes high-mobility group box 1 (HMGB1) protein release, which binds TLR4 on microglia and astrocytes, causing premature microglial activation during embryogenesis, shifting to an M1-like phenotype (pro-inflammatory) and reactive astrogliosis with NF-kB and NLRP3 inflammasome activation leading to a persistent cytokine production and inflammation, which can continue postnatally, causing neurodevelopmental damage.

Likewise, ING proteins have an active role in inflammatory conditions. In fact, ING4 inhibits innate inflammation by boosting NF-κB-mediated activation of IκB promoters, thereby reducing nuclear P65 and pro-inflammatory cytokine levels targeted by NF-κB [67]. In vitro and in animal models, ING4 can reduce lipopolysaccharide-induced upregulation of proinflammatory cytokines by restraining the nuclear translocation and acetylation of the NF-κB pathway by physically interacting with the P65 subunit and causing ubiquitination (by delivering Lys48-linked polyubiquitin to the Lys62 residue of P65) and consequent degradation via a direct interaction with sirtuin1 (SIRT1) [30]. Furthermore, ING1 and ING2 can recruit SIRT1 to the R2 domain of RBP1 and inhibit R2-associated mSIN3A/HDAC1 transcriptional repression, suggesting a possible role similar to that of ING4 in inflammatory conditions [68]. Interestingly, it has been suggested that ING4 and ING3 may play a role in individualized prognostic models to predict early mortality in septic patients [69,70]. Acetylation of histone H4K16, modified by complexes containing ING4, has also been correlated with the expression of myeloperoxidase (MPO) and proteinase 3 (PR3) and more active disease in anti-neutrophil cytoplasmic autoantibody (ANCA)-associated vasculitis [71]. ING1 seems to have a different role in inflammatory conditions as it induces apoptosis via mouse double minute 2 homolog (Mdm2) and initiates an inflammatory response through alternative mechanisms in the absence of the IFN-α/β receptor [72].

### 2.4. Epigenetic Regulation

Reviews summarizing animal and human studies document that parental substance exposure may alter offspring epigenetic regulation and outcomes later in life, including neurodevelopment and mental health [73,74]. Epigenetics is the study of changes in organisms caused by modification of gene expression (rather than alteration of the genetic code itself). In particular, environmental insults that produce oxidative stress, inflammation, or metabolic disruption can perturb DNA methylation, cause histone modifications, and lead to changes in chromatin structure and in payloads of small RNA (miRNA, tRNA fragments, and piRNA) in gametes; some of those perturbations can be transmitted to the embryo and influence early gene regulation [10,75]. The abuse of alcohol and/or drugs during pregnancy may biologically alter offspring phenotypes by causing epigenetic reprogramming during gametogenesis and early embryogenesis. In fact, alcohol metabolism directly interferes with epigenetic regulation, mainly through its effects on one-carbon metabolism, redox balance, and acetyl-CoA availability [74,76].

Alcohol and its metabolic products (e.g., acetaldehyde) reduce folate and S-adenosylmethionine (SAM) availability and increase S-adenosylhomocysteine (SAH) levels, leading to DNA hypomethylation [77,78,79]. Furthermore, alcohol abuse-associated ROS production alters the activity of histone deacetylases (HDACs) and sirtuins, while acetate (metabolic product) increases acetyl-CoA levels, promoting histone hyperacetylation. These changes could be reversible, depending on the amount of intake, duration of exposure, and nutritional status (folate and methionine). Still, long-term use has been associated with neurodevelopmental diseases and possible heritability [52,80,81]. The fetal brain is especially sensitive because in this phase, the DNA methylation patterns are being established, and histone marks are guiding cell fate decisions. Actually, alcohol-related neurodevelopmental diseases are linked to both female and male abuse: several human and rodent studies report altered sperm DNA methylation after paternal exposure to tetrahydrocannabinol (THC), nicotine, and alcohol, targeting genes implicated in neurodevelopment and cardiometabolic regulation [82,83]. Furthermore, some of these changes have been associated with clinical features (e.g., FASD) and transgenerational effects [52]. The epigenetic mechanisms are a major mediator of the effects of addictive drugs on the brain, particularly within the reward circuitry [84]. In fact, addictive drugs (cocaine, alcohol, opioids, nicotine, and amphetamines) induce epigenetic modifications in the brain, especially in the nucleus accumbens and prefrontal cortex, causing persistent changes in brain function and behavior [85,86].

As stated above, the ING family proteins play a prominent role in epigenetic contexts, serving as main regulators, so that HDAC inhibitors targeting ING1 and ING2 are now being produced and tested to target certain cancers using an ING biomarker-guided strategy [87,88]. ING proteins contain conserved plant homeodomain forms of zinc fingers that bind to H3K4me3, consenting them to act as H3K4Me3 readers and target either HDAC (ING1 and 2) or HAT (ING3–5) complexes to alter chromatin by modifying core histones or directing demethylation complexes to DNA. Interestingly, the conformation of the histone tail seems to be able to determine the binding affinity of the docking protein; in particular, the ING protein family tends to prefer the histone H3 tail [89]. In conclusion, ING proteins are endogenous epigenetic guardians that maintain chromatin stability needed for correct neurodevelopment.

### 2.5. Neurodevelopmental Outcomes

Alcohol and drug abuse have been associated with increased risk of neurodevelopmental negative outcomes, leading to cognitive, behavioral and emotional dysfunction, but also physical problems [90]. As stated above, many of these molecules cross the placenta, directly impacting the fetal brain’s development (neurogenesis, neuronal migration, and synaptogenesis) and its signaling pathways [91,92,93,94,95]. Chronic alcohol exposure, as well as to other substances of abuse such as opioids, cocaine, and nicotine, induces oxidative stress, apoptosis, neuroinflammation, and alterations in the expression of neurotrophic genes (e.g., BDNF and CREB) [96,97,98]. Neuronal cells are particularly susceptible to oxidative damage generated by these substances due to their high fatty acid content, elevated metabolic activity, low antioxidant levels, and limited regenerative capacity [47].

In neurons, ROS oxidize proteins, which then gather in the cytoplasm, leading to Aβ plaque formation. Thus, brain cells suffer damage at the genetic and metabolic levels [99,100,101]. These effects are accompanied by epigenetic changes, including DNA methylation, histone modifications, and deregulation of microRNAs [52]. Such a toxic microenvironment could interfere with ING protein function, potentially affecting their expression levels, nuclear localization, or chromatin-binding capacity [12]. In the context of fetal alcohol exposure, extensive evidence demonstrates that ethanol induces persistent epigenetic alterations in neuronal genes through DNA methylation and histone modifications, with long-term consequences on brain development [63]. These changes affect essential neurodevelopmental genes such as neurogenins, Sox family members, and IGF1, many of which are transcriptional targets of ING-associated complexes [102]. Thus, ING proteins may represent previously unrecognized molecular mediators linking substance-induced epigenetic dysregulation to impaired neurodevelopment.

**Figure 1:**
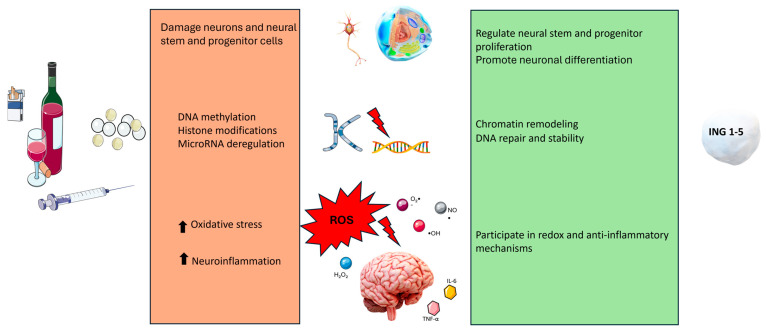
ING proteins versus prenatal abuse of substances: role in neurodevelopment. Gestational alcohol and substance exposure act as upstream epigenetic disruptors and cause oxidative disruption and neuroinflammation, damaging neurons and neural stem and progenitor cells, while ING family proteins regulate neural stem and progenitor proliferation, promote neuronal differentiation, and serve as chromatin readers and genome caretakers whose neurodevelopmental functions are compromised when epigenetic damage exceeds their regulatory capacity. ↑ indicates elevation. Parts of the figure were drawn using pictures from Servier Medical Art and Microsoft PowerPoint 365 Version 2112 (https://www.microsoft.com/microsoft-365, accessed on 11 May 2026). Servier Medical Art by Servier is licensed under a Creative Commons Attribution 3.0 Unported License (https://creativecommons.org/licenses/by/3.0/, accessed on 11 May 2026).

Beyond their canonical role in cancer biology, certain members of the ING family participate in neural stem cell differentiation and neurogenesis (see Figure 1) [12,103]. These activities are partially mediated by interactions with HAT or HDAC complexes, as well as with chromatin-remodeling complexes, including mSin3A, NuRD, and Tip60 [12,104]. As stated before, a shared structural feature of ING proteins is the conserved PHD domain, which can directly bind to H3K4me3, thereby influencing the transcriptional regulation of genes involved in neuronal proliferation and differentiation [32]. Alterations in these processes could contribute to the cellular and brain dysfunction observed in subjects with chronic alcohol exposure. They could intervene in mechanisms regulating cell proliferation and survival in response to damage caused by alcohol. Furthermore, ING1 and ING2 play a role in the establishment of mSIN3a-HDAC complexes, crucial for early embryonic development (a conditional knockout of mSin3A is lethal during early embryogenesis) and the regulation of cell proliferation and survival of both normal and cancerous cells. Experimental work on primary cortical neurons showed that ING1 can be dynamically recruited in response to neuronal activity, suggesting that ING1, traditionally associated with repression and DNA repair, may actively promote neural gene expression, integrating neuronal activity signals with epigenetic mechanisms [15]. In fact, ING1 knockdown led to decreased expression of genes involved in synaptic plasticity, such as Ppp3r1 (a calcineurin regulator).

Meanwhile, ING1 binding to the regulatory region of Ppp3r1 depends on other neuroepigenetic factors, including Piwi-like proteins involved in DNA repair. Interestingly, ING5 exerts its cellular functions through interaction with the HBO1 complex, which plays a crucial role in neural stem cells, leading to the differentiation of neural stem/progenitor cells (NSPCs) into neurons, astrocytes, and oligodendrocytes [37]. HBO1 deficiency reduces H3K14 acetylation, affecting the expression of genes involved in nervous system development and function, ultimately leading to abnormal neurogenesis and impaired brain structural development.

So, ING proteins regulate chromatin structure in the nervous system, influence gene transcription and DNA repair in neurons, and may play roles in neural development and maintenance, as well as in responses to harmful agents such as alcohol and drugs [12]. Furthermore, alterations in ING activity could have implications for neurodegenerative or neurodevelopmental disorders, given its ability to shape the neuronal epigenome.

Unfortunately, despite extensive characterization of ING proteins in cancer biology, their role in neural development remains relatively underexplored. Unfortunately, a major gap in the literature is the scarcity of in vivo studies investigating ING protein function in the developing brain, as most available data derive from tumor cell lines or non-neuronal systems, limiting the direct translatability of these findings to the neurodevelopmental context, particularly under conditions of prenatal environmental stress.

### 2.6. Clinical Relevance and Translational Perspectives

Given their role as epigenetic readers and regulators of chromatin dynamics, ING proteins have been considered candidate biomarkers of cellular stress and epigenetic dysregulation [29,105]. Alterations in ING expression levels, subcellular localization, or interaction with chromatin-modifying complexes could reflect early molecular responses to oxidative stress, inflammation, and toxic environmental exposures.

In particular, the involvement of ING proteins in pathways such as p53 signaling, NF-κB regulation, and DNA damage response suggests that their dysregulation may be detectable in accessible biological samples (e.g., maternal blood, placental tissue, or cord blood) [106,107,108]. Furthermore, ING-associated epigenetic signatures, such as changes in histone acetylation/deacetylation or transcriptional profiles of target genes, may provide indirect but measurable indicators of disrupted chromatin regulation during fetal development [109]. However, it should be noted that no studies to date have directly validated ING proteins as biomarkers in prenatal exposure settings, and their specificity and sensitivity relative to established oxidative stress or inflammatory markers remain to be determined.

Meanwhile, ING-dependent molecular alterations may also have potential utility in early diagnosis or risk stratification, as prenatal exposure to alcohol and drugs is known to induce epigenetic and inflammatory changes before the onset of clinically detectable neurodevelopmental abnormalities [53]. In this framework, ING-related chromatin and transcriptional signatures could contribute to identifying individuals at increased risk.

Another interesting setting is the possible therapeutic application of current knowledge on ING proteins. As suggested by other authors (mainly in papers related to cancer), the involvement of ING proteins in chromatin remodeling and stress response pathways raises the possibility that they could be explored as therapeutic targets, particularly through modulation of their associated epigenetic complexes [110].

In fact, ING1 and ING2 are closely linked to histone deacetylase (HDAC) complexes, while ING3–5 interact with histone acetyltransferase (HAT) complexes such as Tip60 and HBO1 [14,27,39]. Pharmacological agents targeting these pathways, including HDAC inhibitors and modulators of histone acetylation, are already under investigation in oncology and may have broader applicability in conditions characterized by epigenetic dysregulation. Nevertheless, direct targeting of ING proteins themselves may be challenging, and indirect modulation of their interacting partners is likely to represent a more feasible approach. Importantly, any therapeutic application in pregnancy would require careful evaluation of safety, timing, and developmental specificity.

## 3. Discussion

**Figure 2:**
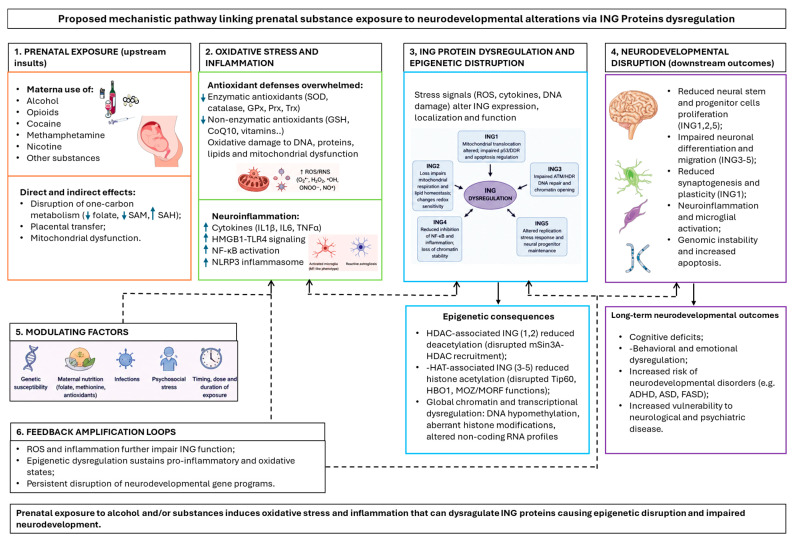
Proposed mechanistic pathway linking prenatal substance exposure to neurodevelopmental alterations via ING proteins dysregulation. ↑ indicates elevation. ↓ indicates reduction. Parts of the figure were drawn using pictures from Servier Medical Art and Microsoft PowerPoint 365 Version 2112 (https://www.microsoft.com/microsoft-365, accessed on 11 May 2026). Servier Medical Art by Servier is licensed under a Creative Commons Attribution 3.0 Unported License (https://creativecommons.org/licenses/by/3.0//, accessed on 11 May 2026).

In this narrative review, we summarize the literature evidence on the potential association between ING family protein expression and neurodevelopmental alterations in newborns exposed to prenatal alcohol and/or drugs (see Figure 2 for a schematic model summarizing the proposed mechanistic pathway).

This review has clearly shown the established role of ING proteins in chromatin remodeling, DNA damage response, and p53 signaling, as well as the robust evidence linking prenatal exposure to alcohol and drugs with oxidative stress, inflammation, epigenetic dysregulation and neurodevelopment. Meanwhile, only indirect evidence has linked the involvement of ING proteins in oxidative stress responses, mitochondrial function, and inflammatory signaling. Oxidative stress is a primary upstream insult, while inflammation is a second major pathogenic axis, with ING proteins acting as stress response modulators and epigenetic regulators. Furthermore, emerging data support the indirect evidence that ING members (e.g., ING1 and ING5) play specific roles in neuronal function and neurodevelopment. Based on what has been demonstrated directly and indirectly, we propose the new hypothesis of a role of ING proteins as mediators linking prenatal alcohol or drug exposure to neurodevelopmental outcomes. This role is possibly linked to the potential disruption of ING-dependent chromatin regulation by substance-induced epigenetic alterations.

ING proteins regulate DNA repair and respond to cellular stress signals, including those that may compromise neuronal development and genomic stability (e.g., due to prenatal exposure to toxins). The fact that ING1 responds to neuronal activity and modulates genes critical for synaptic plasticity (like Ppp3r1) suggests a possible role in regulating neuronal development and synapse maturation [15]. Furthermore, ING5, with its roles in stem cells and HAT complexes, could influence neural differentiation and neurogenesis/regenerative capacity. While, on the one hand, alcohol and drug prenatal exposure may elicit neurodevelopmental diseases through their impact on mitochondria and oxidative stress, neuroinflammation and epigenetics (DNA methylation, histone modifications, chromatin structure, and small RNA), on the other hand, ING proteins could play the important role of guardians protecting from ROS and inflammatory excesses and regulating epigenetic changes to avoid gene and cellular aberrations.

Despite the extensive literature on ING proteins as tumor suppressors, their role in neural development (especially ING4 and ING5) remains experimentally poorly characterized.

Many ING studies focus on tumor cell lines or cancer models rather than primary neurons or in vivo brain models. There are a few studies directly linking prenatal exposure (e.g., alcohol and drugs) to ING changes during neuroembryonic development, a promising and relatively unexplored research area.

This study has several limitations. As this is a narrative review, no systematic protocol, risk-of-bias assessment of included studies or quantitative synthesis has been carried out, so the evidence synthesis may not capture the full scope of the available literature. Furthermore, to the best of our knowledge, this is the first study that links specifically ING proteins to neurodevelopment in newborns from mothers who abuse alcohol and/or drugs, so limited direct evidence is available in the literature.

Importantly, direct experimental evidence linking prenatal exposure to alcohol or drugs with alterations in ING protein expression or function during neurodevelopment is currently lacking. Furthermore, the functional roles of individual ING proteins appear to be highly context-dependent. This may be due to the heterogeneity in experimental models (mostly cell lines and animal models), exposure paradigms, and outcome measures, which make it difficult to draw consistent conclusions across studies. These limitations highlight a critical gap in the field and underscore the need for targeted in vivo and longitudinal studies to clarify whether ING proteins play a causal role in mediating the neurodevelopmental effects of prenatal exposure to alcohol and drugs.

## 4. Materials and Methods

In January and February 2026, a comprehensive literature search was performed across PubMed, Scopus, and Web of Science. The search strategy employed combinations of Boolean operators (AND/OR) with the following keywords: (“ING proteins” OR “Inhibitor of Growth”) AND (“neural development” OR “neurogenesis”); (“ING proteins” OR “Inhibitor of Growth”) AND (“alcohol” OR “drugs” OR “substance abuse”); and (“ING proteins” OR “Inhibitor of Growth”) AND (“epigenetics” OR “oxidative stress” OR “neuroinflammation”).

The inclusion criteria were limited to (1) original research articles published in English, and (2) studies specifically investigating the role of ING proteins in neural development in relation to prenatal exposure to alcohol or drugs. The exclusion criteria included letters, editorials, case reports, and conference abstracts to ensure the inclusion of peer-reviewed, primary data only.

The screening process was conducted independently by S.T. and M.F., who evaluated titles and abstracts; any discrepancies were resolved by a third party (G.F.). From an initial pool of 107 records, 55 were selected for full-text assessment, and 49 were ultimately included. The quality of the included studies was independently assessed by the authors, focusing on methodological soundness, sample size, and the clarity of the reported results. Only studies with a clear experimental design and reproducible findings were included in the discussion. To improve clarity, the results are organized into four interconnected mechanistic domains (oxidative stress, inflammation, epigenetic regulation, and neurodevelopmental outcomes) progressing from upstream insults to downstream phenotypic effects.

## 5. Conclusions

While ING proteins are well established as regulators of chromatin dynamics, DNA damage response, and cellular stress pathways, their role in neurodevelopment, particularly in the context of prenatal environmental insults, remains incompletely understood. The available evidence suggests that ING proteins are positioned at the intersection of key mechanisms implicated in neurodevelopmental toxicity, including oxidative stress, inflammation, and epigenetic dysregulation. However, it is important to emphasize that direct experimental evidence linking prenatal substance exposure to alterations in ING protein function during neurodevelopment is currently lacking, and most of the proposed connections are based on indirect or extrapolated findings. Therefore, the hypothesis that ING proteins act as mediators of substance-induced neurodevelopmental alterations should be considered exploratory and hypothesis-generating.

Future research should focus on investigating ING protein expression and function in relevant in vivo neurodevelopmental models and in assessing their modulation following prenatal exposure to specific substances. These studies may clarify whether ING proteins contribute to the molecular vulnerability of the developing brain to chronic toxic insults. Furthermore, ING proteins’ potential role as biomarkers or therapeutic targets should be evaluated in longitudinal human studies. Although direct experimental evidence is lacking, our hypothesis is that exposure to drugs of abuse may disrupt ING proteins’ expression or function, leading to aberrant epigenetic responses to toxic insults and, finally, to negative neurodevelopmental outcomes.

## Figures and Tables

**Table 1:** ING proteins’ molecular mechanisms and biological functions. All ING proteins contain a C-terminal PHD finger that specifically binds trimethylated lysine 4 on histone H3 (H3K4me3), acting as epigenetic adaptors that read H3K4me3 and translate chromatin state into DNA damage signaling, transcriptional control, senescence, or apoptosis. DDR, DNA damage response; HAT, histone acetyltransferase complex; HDAC, histone deacetylase; NLS, nuclear localization sequence; PHD, plant homeodomain.

ING Protein	Structural Domains and Binding Specificity	Major Interacting Complexes	Epigenetics	Signaling Pathways	Functional Implications	Pathological Implications
ING 1	PHD finger, NLS, and PCNA-interacting region	mSin3A–HDAC1/2, p53, and PCNA	Promotes histone deacetylation at active chromatin; stabilizes p53 occupancy at target promoters	p53 pathway, DNA damage response (DDR), and cell-cycle checkpoints	G1/S arrest, senescence, and apoptosis in response to genotoxic stress	Frequently downregulated or mislocalized in solid tumors; loss contributes to genomic instability and impaired senescence
ING 2	PHD finger, leucine zipper, and PI(5)P-binding motif	mSin3A–HDAC, p53, p300, and phosphoinositide signaling components	Couples’ nuclear lipid signaling to chromatin repression; HDAC-mediated transcriptional silencing	p53 signaling, oxidative stress response, and oncogene-induced stress	Apoptosis, stress-induced growth arrest, metabolism and lipid homeostasis, and cell migration	Reduced expression in cancers; altered nuclear lipid–chromatin crosstalk may promote tumor progression
ING 3	PHD finger	Tip60/NuA4 HAT complex	Facilitates histone H4 acetylation and chromatin relaxation at repair and transcription sites	DDR, homologous recombination, and transcriptional activation	DNA repair efficiency, differentiation, and controlled proliferation	Dysregulation linked to defective DNA repair and increased mutational burden
ING 4	PHD finger and coiled-coil domain	p53, NF-κB (RelA), HBO1 HAT complexes, and MOZ/MORF HAT complexes	Context-dependent chromatin modulation; favors transcriptional repression of inflammatory genes	p53 activation, NF-κB inhibition, and hypoxia/angiogenesis pathways	Apoptosis, anti-inflammatory signaling, and inhibition of angiogenesis	Loss associated with aggressive tumors, enhanced inflammation, and pro-tumorigenic microenvironment
ING 5	PHD finger and nuclear localization motifs	p300/CBP, HBO1 HAT complex, and MOZ/MORF HAT complex	Promotes H3 and H4 acetylation during DNA replication and repair	p53 acetylation and replication stress response	Cell-cycle regulation, genomic stability, and apoptosis	Downregulation contributes to replication stress tolerance and cancer cell survival

## Data Availability

Data supporting the reported results can be found at PubMed (https://pubmed.ncbi.nlm.nih.gov, accessed on 11 May 2026), Scopus (https://www.sciencedirect.com, accessed on 11 May 2026) and Web of Science (https://www.webofscience.com/wos/woscc/basic-search, accessed on 11 May 2026).

## Abbreviations

The following abbreviations are used in this manuscript: DNA damage response (DDR), fetal alcohol spectrum disorders (FASDs), glutathione (GSH), high-mobility group box 1 (HMGB1), histone acetyltransferase (HAT), histone deacetylase (HDAC), inhibitor of growth (ING), nuclear factor erythroid 2-related factor 2 (NRF2), nuclear factor-kappa B (NF-kB), nuclear localization sequence (NLS), plant homeodomain (PHD), proliferating cell nuclear antigen (PCNA), reactive nitrogen species (RNS), reactive oxygen species (ROS), S-adenosylmethionine (SAM), sirtuin1 (SIRT1), superoxide dismutases (SOD), tetrahydrocannabinol (THC), and trimethylated lysine 4 on histone H3 (H3K4me3).
